# Optical Projection Tomography Using a Commercial Microfluidic System

**DOI:** 10.3390/mi11030293

**Published:** 2020-03-11

**Authors:** Wenhao Du, Cheng Fei, Junliang Liu, Yongfu Li, Zhaojun Liu, Xian Zhao, Jiaxiong Fang

**Affiliations:** 1 Center for Optics Research and Engineering, Shandong University, Qingdao 266237, China; duwenhaojob@126.com (W.D.); 18769780795@126.com (C.F.); zhaoxian@sdu.edu.cn (X.Z.); jx@sdu.edu.cn (J.F.); 2 School of Information Science and Engineering, Shandong University, Qingdao 266237, China; julysrain@yeah.net (J.L.); zhaojunliu@sdu.edu.cn (Z.L.); 3 Key Laboratory of Infrared Imaging Materials and Devices, Shanghai Institute of Technical Physics, Chinese Academy of Sciences, Shanghai 200083, China

**Keywords:** microfluidics, OPT, microscope, lab-on-a-chip, focal plane scanning

## Abstract

Optical projection tomography (OPT) is the direct optical equivalent of X-ray computed tomography (CT). To obtain a larger depth of field, traditional OPT usually decreases the numerical aperture (NA) of the objective lens to decrease the resolution of the image. So, there is a trade-off between sample size and resolution. Commercial microfluidic systems can observe a sample in flow mode. In this paper, an OPT instrument is constructed to observe samples. The OPT instrument is combined with commercial microfluidic systems to obtain a three-dimensional and time (3D + T)/four-dimensional (4D) video of the sample. “Focal plane scanning” is also used to increase the images’ depth of field. A series of two-dimensional (2D) images in different focal planes was observed and compared with images simulated using our program. Our work dynamically monitors 3D OPT images. Commercial microfluidic systems simulate blood flow, which has potential application in blood monitoring and intelligent drug delivery platforms. We design an OPT adaptor to perform OPT on a commercial wide-field inverted microscope (Olympusix81). Images in different focal planes are observed and analyzed. Using a commercial microfluidic system, a video is also acquired to record motion pictures of samples at different flow rates. To our knowledge, this is the first time an OPT setup has been combined with a microfluidic system.

## 1. Introduction

Three-dimensional imaging has become an effective tool for biomedical research. However, a gap between macroscopic imaging technology and microscopic imaging technology led to an inability to observe samples of certain sizes. This gap was filled by optical projection tomography (OPT) technology.

OPT technology enables three-dimensional imaging of samples that are 1–10 mm in size. Samples of this size are too large for confocal imaging, and too small for magnetic resonance imaging (MRI), but most vertebrate embryos are in this size range [1,2]. OPT microscopy is especially suitable for imaging samples whose size lies between 0.5 mm and 10 mm. It is difficult to use confocal microscopy to produce high-quality images at depths greater than 0.5 mm, and the resolution of nuclear magnetic resonance (NMR) imaging is too low to observe all tissues and organs. OPT is also capable of utilizing many colored and fluorescent dyes that were developed for tissue-specific or gene-specific staining, which is important to three-dimensional observations of specific tissues because it allows a computer to automatically determine the three-dimensional structure of the target tissue. Many universities [3,4,5,6,7,8,9,10,11] have adopted OPT systems to help them do biomedical research.

To improve OPT’s imaging quality and imaging speed, many universities and research institutions have focused on basic research on imaging technology. Optimizing the OPT imaging technology has attracted researchers’ interest. The OPT technology has also been under continuous development, including the improvement of the system itself, the improvement of algorithms, and expansion to other new imaging technologies. Trull, van der Horst et al. [12] applied the point transfer function of the lens to an iterative reconstruction algorithm, and proposed a new optical tomography reconstruction technique with filtered back projection. Correia, Lockwood et al. [13] used an iterative algorithm to reconstruct a sparsely sampled OPT dataset that significantly reduces the minimum acquisition time and light dose while maintaining the image quality. To image non-transparent tissue in vivo, Marcos-Vidal, Ancora et al. [14] applied the near-infrared band (1300–1400 nm) to OPT imaging. Compared with visible light, near-infrared light can increase the penetration depth and reduce the effects of autofluorescence and scattered light. The mechanism uses lasers in different wavelength bands as light sources to evaluate the imaging characteristics and the advantages of different wavelength bands. There are also several studies [15,16,17,18,19,20,21,22] that focus on improvements to OPT systems.

In recent years, many studies have been conducted to improve the performance of OPT [23,24,25]. However, in most of these studies, the effects of stray light are not considered, and the main work focuses on transparent objects or in vitro imaging after drug cleaning. In order to meet the needs of OPT imaging of live samples (non-transparent tissue, dynamic imaging), there is an urgent need to improve OPT’s resolution and imaging speed. Living tissue samples cannot be pre-treated, so a higher resolution is required compared with in vitro imaging, and we need to minimize the effects of stray light to improve the image quality. To image live samples, improving the resolution is a significant challenge. However, in order to obtain projection images, the depth of field (DOF) needs to cover at least half of the specimen [26]. As a result, there is a trade-off between image resolution and DOF [27]. For large samples, a low numerical aperture (NA) lens is used to obtain a large DOF while sacrificing resolution. The DOF can also be extended by axially scanning the focal plane of the objective lens through the sample [28]. Using this method, a high-NA objective lens can be used to simultaneously obtain a large DOF and a high resolution.

In this paper, we designed an OPT adaptor to perform optical projection tomography on a wide-field inverted microscope. A commercial microfluidic system was used to observe the sample in flow mode. A series of images in different focal planes was observed and analyzed. An algorithm was applied to defocus the images in different positions. A video of the sphere was recorded at a specific flow rate to illustrate the dynamic motion of the samples. The advantage of the system is that it uses a commercial microfluidic system to enable the observation of images at different flow rates.

## 2. Experiment and Simulation

### 2.1. Experimental Setup

Our work was performed using an inverted microscope (model number: olympusix81). A high-speed camera (ImagEM X2 EM-CCD camera C9100-23B) was used in our microscopy system. The number of effective pixels was 512 (H) × 512 (V), and the pixel size was 16 μm (H) × 16 μm (V). The camera is characterized by a fast imaging speed, extremely high quantum efficiency in the effective wavelength range (up to 90% or more), and an excellent signal-to-noise ratio under deep cooling conditions. The commercial microfluidic system has three components. The pump can be used to adjust the flow rate, and the fluidic channel is etched and consists of three parallel aisles. The microunit chip holder is designed to fit the stage and hold the channels. The microfluidic system was modified to connect to the stepper motor so that the channels can rotate when the sample flows.

We designed an OPT plate. The OPT adaptor was designed to fit into the aperture of a common 160 × 110 mm microscope stage. It consists of three main components that were fabricated using aluminum materials. To provide for controlled adjustment of the tilt angle, we separated the two plates. An aluminum dowel is seated in grooves on each plate at one end. At the other end, there is a fine thread adjustment screw (P25SB100L, Thorlabs Inc, Newton, NJ, USA) that allows the distance between the two plates to be adjusted. The stepper motor is mounted directly onto the side of the sample chamber, with its axle connecting the channels in the microfluidic system. An aluminum mounting port is attached to the motor’s axle to allow the microfluidic channels that contain the sample to be easily mounted in the chamber. When we rotate the motor, the microfluidic channels will rotate together. So, we can take pictures of every aspect of our sample. Using images taken from different angles of the sample, we can reconstruct three-dimensional (3D) images of our sample. These images show every detail of the sample (e.g., a colloidal particle). With the high-precision stepper motor (an NM08AS-T4-MC04-HSM8 Stepper motor with a home sensor, NEMA size 08 × 33 mm, single shaft), we can rotate the sample and obtain two-dimensional (2D) projection information at different angles. Then, the 2D projection information is used to reconstruct 3D information about the sample. The filtered back projection algorithm is used in the reconstruction process.

**Figure 1:**
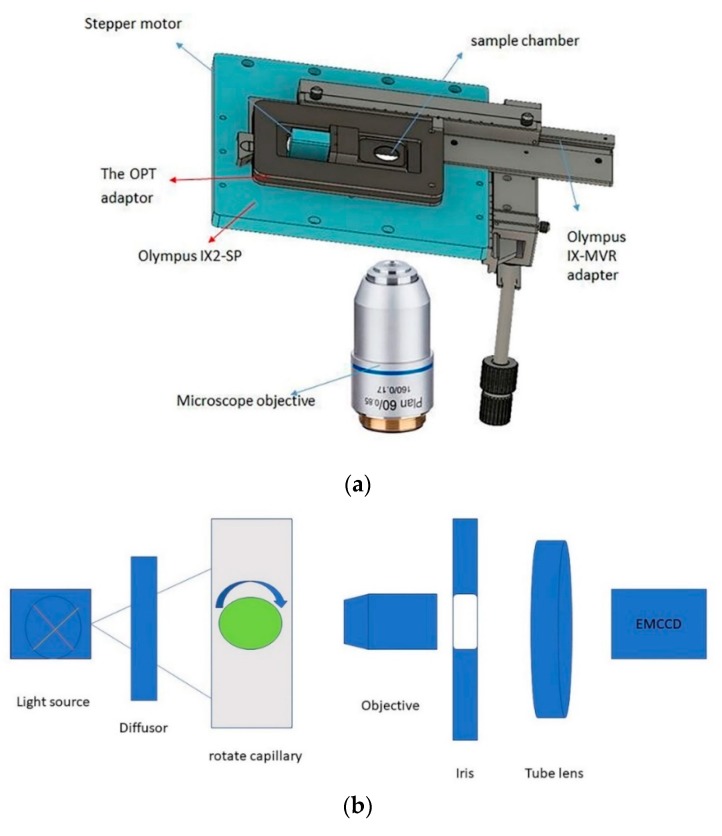
(**a**) A microscope platform that can observe samples at different angles; (**b**) an overview of the experimental optical projection tomography system. A mercury lamp is used to pass trans-illumination through a diffusor. The iris can adjust the numerical aperture (NA) of the objective lens. The light converges through the tube lens. OPT, optical projection tomography.

As Figure 1 illustrated, we provide an overview of the experimental optical projection tomography system. We combined our microscope platform with a commercial microfluidic system so that we could observe samples in flow mode. A pump was used to control the flow rate. First, a solution of magnetic polystyrene microspheres in ethanol (at different concentrations) was prepared as a sample. The diameter of the microspheres was 19 μm. The microfluidic channels were etched so that we could observe the flow of the microspheres. The polystyrene microspheres, at different flow rates and solution concentrations, were observed using pump-controlled flow rates. Magnetic fields of different strengths were generated by an alternating current power source and a self-made copper wire coil to guide the flow of the polystyrene microspheres.

### 2.2. Simulated Method

In this part, we introduce a program by which to compute an image. In the following, the bold letters represent two-dimensional vectors. **T**(**m**) is the spectrum of the specimen, and **t**(**x**) is the transmission of the specimen. **P*_o_***(ξ) is the pupil function of the objective back focal plane (BFP), and **P*_c_***(ξ) corresponds to part of the condenser’s front focal plane (FFP). We use **F** to denote the Fourier transform. **P*_c_*** is the intensity of the illumination pupil, and **P*_o_*** indicates the amplitude of the objective pupil. The **P*_o_***(ξ) filters the diffraction orders, and hence acts as a low-pass filter. The filtered power spectrum |***T***(***m***)***P_o_***(***m***)|^2^ denotes the intensity distribution of the objective back focal plane. The recorded intensity, ***I*** = |***F_m_***^−1^***T***(***m***)***P_o_***(***m***)|^2^, is the squared magnitude of the image’s amplitude. Therefore, the total image intensity [29,30] is given as

$$I(x)={\int \left|Pc(\xi )\right|}^{2}I\varepsilon (x)d\xi ,$$

(1) $$I(x)={\int \left|Pc(\xi )\right|}^{2}\iint_{}T(m1-\xi )T^{*}(m2-\xi )P_{o}(m1){P_{o}}^{*}(m2)e^{2\pi i(m1-m2)x}dm1dm2d\xi$$

With quasi-monochromatic partially coherent illumination, the 2D image recorded by a microscope, according to the sum-over-source algorithm [29], is as follows:

(2) $$I(x,y)={\iint_{}S(\xi ,\eta )\left|F\left[T(f_{x}-\xi ,f_{y}-\eta )P(f_{x},f_{y})\right]\right|}^{2}d\xi d\eta$$

We use I(**x**,**y**) to indicate the image intensity, and S(ξ,**η)** to denote the source intensity’s distribution. ***T***(***f_x_***,***f_y_***) indicates the spectrum of the object, and ***P***(***f_x_***,***f_y_***) corresponds to the amplitude of the imaging pupil. The distribution of the effective refractive index is given by n(x,y,z) =$\sqrt{\epsilon \;\left(x,\;y,\;z\right)}$. Therefore, the optical path difference profile [24] is

(3) $$OPD(x,y)=\int \frac{2\pi }{\lambda }\left[n(x,y,z)-n_{w}\right]dz=\int \frac{2\pi }{\lambda }\left[\sqrt{\varepsilon (x,y,z)}-\sqrt{\varepsilon_{\omega }}\right]dz$$

The specimen transmission function is given by

(4) t(x,y)=exp[iOPD(x,y)].

## 3. Results and Discussion

### 3.1. Experimental Procedure

**Figure 2:**
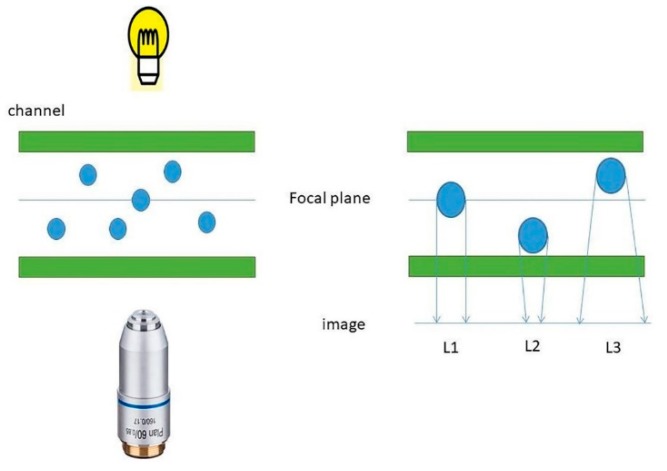
Analysis of the projection of different focal planes of the same microspheres.

We combined our microscope platform with a commercial microfluidic system so that we could observe samples in flow mode. Through experimental observations, images of polystyrene microspheres with different focal planes were obtained and compared with the simulated images. Images and videos of microfluids at different flow rates were also obtained. Under the illumination of a bright field source, the dimensions of the same polystyrene microspheres were different in different focal planes. As illustrated in Figure 2, when the focal plane was located in the centre of the sphere, we obtained a clear image (i.e., the image is in focus). When the focal plane was located above the sphere, the size of the image (L2) was different from that of the in-focus image (L1). A similar situation occurred when the sphere was located at the top. This analysis shows the relationship between projection size and focal plane. When the focal plane was located at the bottom, the image with the largest projection size was obtained. As the focal plane gradually moved up, the projection size in the image was gradually reduced.

### 3.2. Experimental Results

The focal plane scanning technique can be used to obtain information at different depths (Z-axis sizes) by moving the position of the focal plane in a sample. The range of depths of a clear image can be obtained. This is called the depth of field (DOF), which can be effectively improved by reducing the numerical aperture of the objective lens. This method can be applied to traditional OPT imaging by placing an aperture directly behind the objective lens, which sacrifices optical resolution to achieve a greater depth of field. The focal plane scanning method can extend the DOF without sacrificing the optical resolution of OPT imaging. In our experiment, a total of 125 images was obtained. The Z-axis indentation between each image was 0.47 μm, the number of effective pixels was 512 (H) × 512 (V), the pixel size was 16 μm (H) × 16 μm (V), the magnification was 32×, and the real space interval between pixels was 500 nm, so the spatial field of view of the entire picture was 256 μm × 256 μm. As the Figure 2 illustrated, the focal plane scanning results of polystyrene microspheres were analyzed.

**Figure 3:**
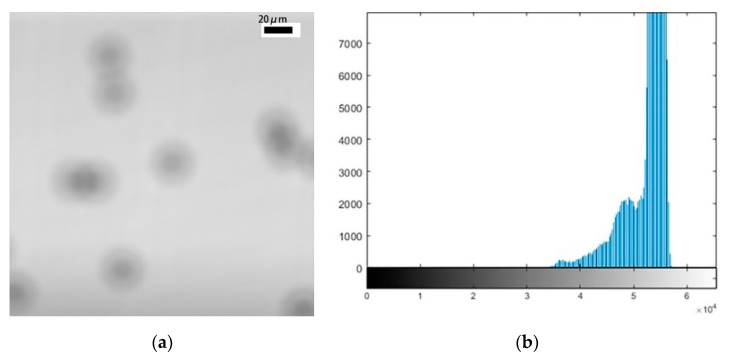
An image of microspheres when the focal plane is at the bottom and the corresponding histograms. (**a**) The image of microspheres; (**b**) the corresponding histograms.

**Figure 4:**
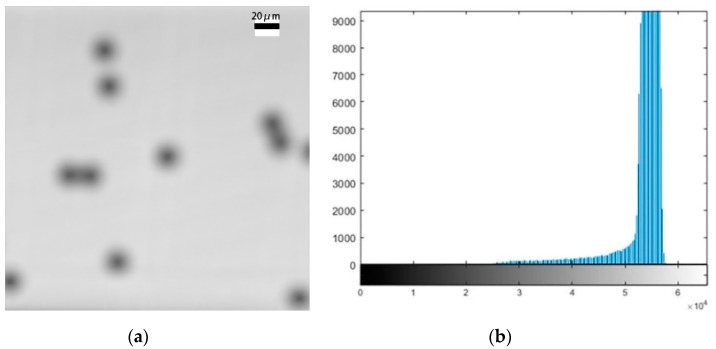
An image of microspheres when the focal plane is at the top and the corresponding histograms. (**a**) The image of microspheres; (**b**) the corresponding histograms.

**Figure 5:**
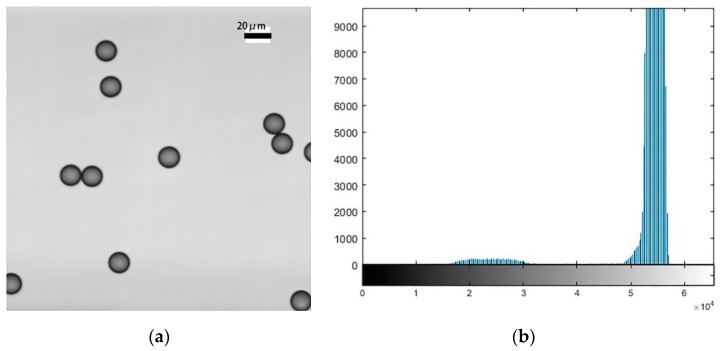
An in-focus image of polystyrene microspheres and the corresponding histograms. (**a**) The image of polystyrene microspheres; (**b**) the corresponding histograms.

Figure 3 and Figure 4 show maximum out-of-focus images of the polystyrene microspheres and their corresponding histograms. Figure 3 shows that the size of the microspheres is larger when the focal plane is at the bottom; this phenomenon was analyzed in Figure 2. The experimental results are consistent with the theoretical analysis. Comparing Figure 3 with Figure 4, we can see that the image is clearer when the focal plane is at the top. We also can see the reason for this from their respective histograms. The pixel distribution has a higher concentration when the focal plane is at the top. A pixel distribution with a higher concentration provides a better image contrast. Figure 5 shows a clear image when the focal plane was located at the centre of the sphere.

From the above experimental data, it can be found that the size of the spheres was significantly larger (larger than the actual size) when the focal plane was located above the small sphere, and the imaging size of the spheres was smaller than the actual size when the focal plane was located below the small sphere. This result is consistent with the previous analysis.

**Figure 6:**
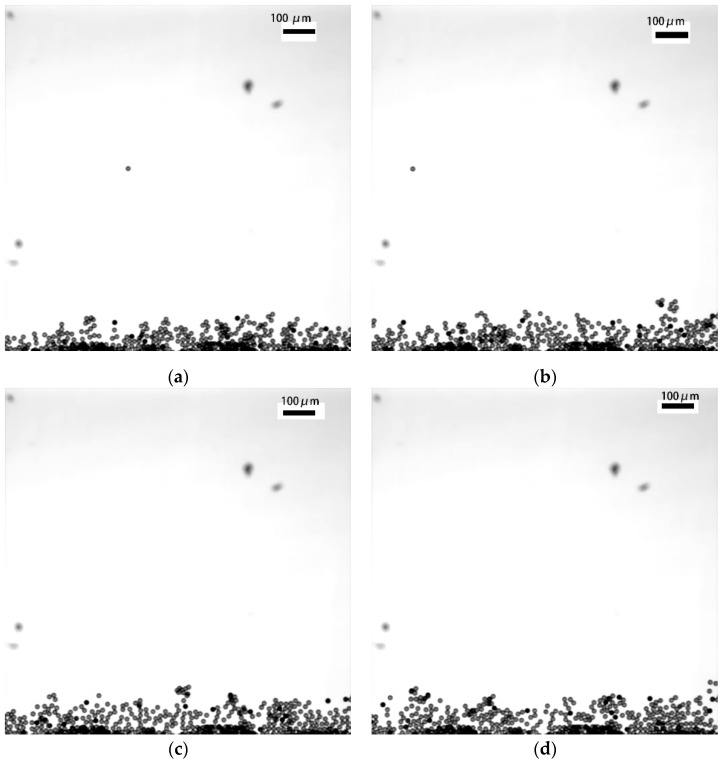
Images of four frames from the video. (**a**) An image of the 100th frame of the video of the microspheres; (**b**) an image of the 200th frame of the video of the microspheres; (**c**) an image of the 300th frame of the video of the microspheres; (**d**) an image of the 400th frame of the video of the microspheres.

The microspheres in flow mode were also observed. The flow rate can be controlled using a pump. At a flow rate of 1 µL/min, video stream data on the polystyrene microspheres were obtained using the modified system. The diameter of the microspheres was 19 µm. A total of 4432 frames was obtained in our video. As shown in Figure 6, four frames were selected to illustrate the motion of the microspheres.

As shown in Figure 6, the modified system can be used to observe the dynamic changes in microspheres. OPT requires the DOF of the lens to cover at least half of the sample. There is a trade-off between obtaining a high resolution with a high-NA lens and obtaining a large DOF with a low-NA lens. The DOF of a high-NA objective lens can be extended by scanning its focal plane through the sample. We call this extended DOF image a “pseudoprojection”. Images reconstructed from these pseudoprojections have an isometric resolution, which may be identical to the lateral resolution of the high-NA objective lens. The DOF of a high-NA objective lens can be extended by scanning the focal plane through the sample. This can overcome the constraint on conventional OPT, which requires a low-NA objective lens in order to obtain a large DOF.

**Figure 7:**
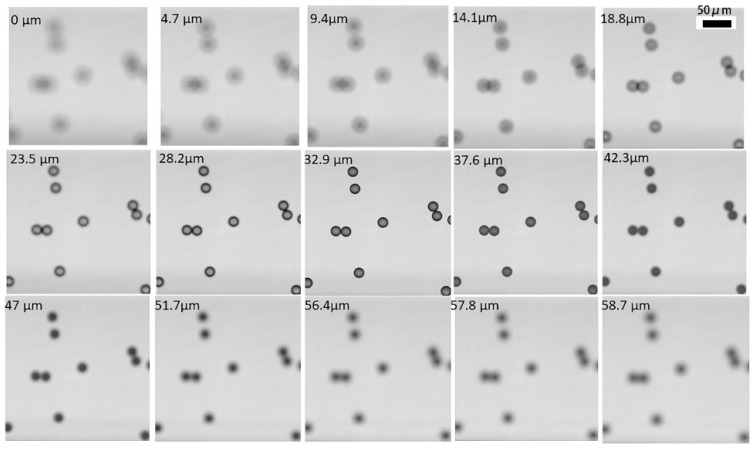
The results of the focal plane scanning.

The focal plane gradually moves up; so, we set the distance to 0 μm when the focal plane was located at the bottom. The distance from the focal plane to the bottom is marked in the upper left corner of each image in Figure 7. A total of 125 images was obtained. The focal plane indentation distance between each image was 0.47 μm, the number of effective pixels was 512 (H) × 512 (V), the pixel size was 16 μm (H) × 16 μm (V), the magnification was 32×, and the interval between pixels was 500 nm, so the spatial field of view of the entire picture was 256 μm × 256 μm. As illustrated in Figure 7, the images in different focal planes of a single sample are different. Images with different focal planes reflect the cross sections of the same object in different dimensions.

In Table 1, ‘NA’ represents the numerical aperture of the objective lens. We used a 4× objective lens with an NA of 0.16 (Olympus). Although the DOF of this objective lens is only about 16.27 μm, our method applies “focal plane scanning” to the OPT system. In contrast, for conventional OPT, a lens with an NA of about 0.025 is needed to achieve a DOF of 1 mm. The “focal plane scanning” method extends the imaging’s depth of field without sacrificing resolution.

### 3.3. Simulation Results

**Figure 8:**
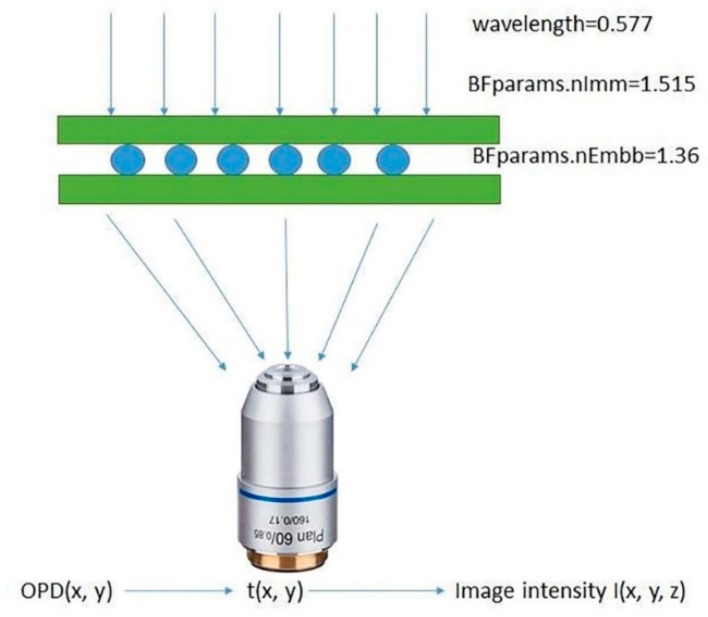
The schematic of the image’s computation. OPD, optical path difference.

As Figure 8 illustrated, we provide an overview of a simulation program. A matlab program was used to compute our image. The first thing we needed to do was set the refractive index and the wavelength of the light source. The refractive index of the polystyrene microspheres was set to 1.55. The alcohol solution of the polystyrene microspheres flowed between two glasses, the refractive index of the glasses was set to 1.515, and the refractive index of the alcohol was 1.36. The illumination wavelength of the microscope was 0.577 μm, and the diameter of the spheres was 19 μm. These parameters were used to calculate the optical path difference (OPD) (x,y). The specimen’s transmission function is given by t(x,y) = exp [iOPD(x,y)], so we can compute the image from the specified transmission. The compute image function accepts a 2D matrix (representing the specimen’s transmission) as an input and computes the final intensity image, which will be a 3D matrix if the grid axis along the axial direction is a vector. The specific steps of the simulation are as follows: (1) set the parameters of the microscope and the object; (2) choose a small simulation region for a reasonable runtime; (3) set the parameters of the bright field microscope and the image; and (4) compare the simulated images with the originals.

**Figure 9:**
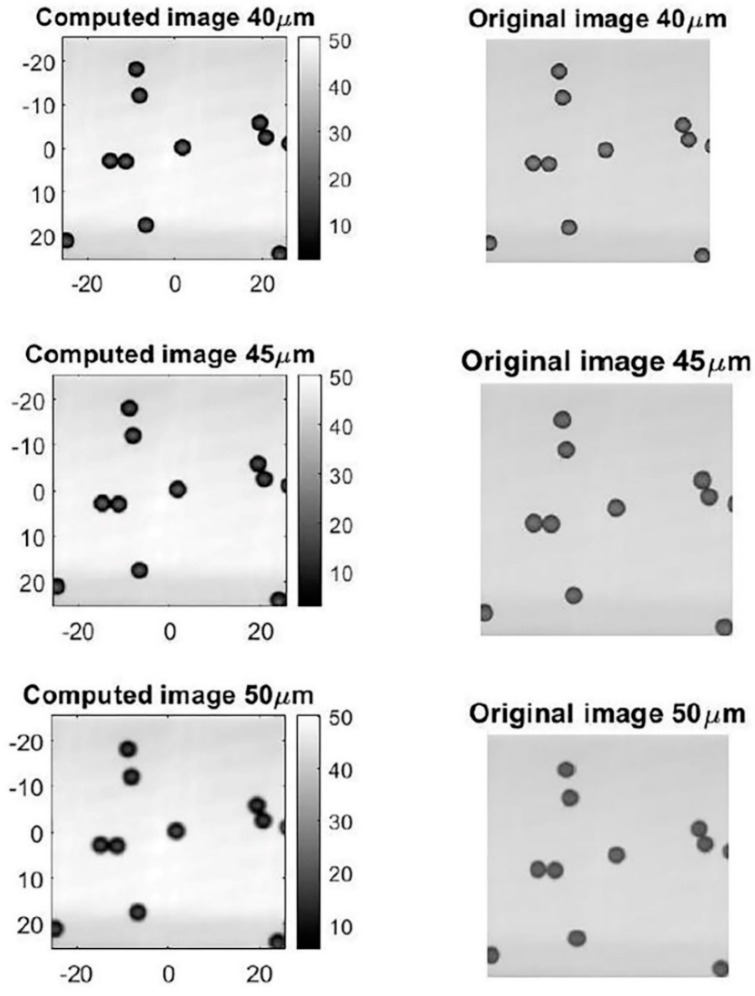
The simulated pictures and the corresponding experimental pictures.

Images in a “defocused state” were simulated using an algorithm and compared with the real images acquired during our experiment. We also marked the focal plane position in terms of distance to the bottom. As shown in Figure 9, the algorithm was able to compute the images when the focal plane was located in different positions. The results of the simulation show that our program can be used to reliably compute images and simulate images on different focal planes.

## 4. Conclusions

In this study, an OPT system was constructed using an inverted microscope. The combination of a commercial microfluidic system and our microscope platform can be used to observe samples in flow mode. OPT requires the imaging’s depth of field to cover at least half of the sample. However, in the traditional method, we need to reduce the numerical aperture of the objective lens by placing a pinhole. This significantly lowers the resolution of the image. To optimize this trade-off, the “focal plane scanning” technology was applied to increase the imaging’s depth of field, and a series of focal plane scanning images was obtained and analyzed. The simulation and image calculations were performed on defocused images with different Z-axis sizes, and the computed images were compared with real images. As a 3D imaging tool, OPT plays a crucial role in many applications. A microfluidic system can be used to observe microsamples in flow mode. A combination of OPT and microfluidics enables 3D dynamic monitoring of microsamples. Using this technology, we can obtain three-dimensional and time (3D + T)/four-dimensional (4D) images of samples with a special size. This method is innovative and, in the future, may help us to observe the complex changes that occur in the microworld. In this study, images taken on different focal planes were observed and analyzed. The simulated images were also compared with real images. A video was recorded to show the dynamic changes of the microspheres. This work is of significance to improving the resolution of OPT technology and the dynamic monitoring of microenvironments.

## Figures and Tables

**Table 1:** The resolution and depth of field (DOF) of the optical projection tomography (OPT) system. NA, numerical aperture.

	Traditional Method with an NA of 0.13	Traditional Method with an NA of 0.055	Traditional Method with an NA of 0.025	Our Method
Resolution	3.315 μm	5.6 μm	7.7 μm	2.58 μm
DOF	0.04 mm	0.21 mm	1 mm	1 mm
